# Exploration of the link between COVID-19 and gastric cancer from the perspective of bioinformatics and systems biology

**DOI:** 10.3389/fmed.2024.1428973

**Published:** 2024-09-20

**Authors:** Xiao Ma, Tengda Huang, Xiaoquan Li, Xinyi Zhou, Hongyuan Pan, Ao Du, Yong Zeng, Kefei Yuan, Zhen Wang

**Affiliations:** Division of Liver Surgery, Department of General Surgery and Laboratory of Liver Surgery, and State Key Laboratory of Biotherapy, West China Hospital, Sichuan University, Chengdu, China

**Keywords:** bioinformatic analysis, hub genes, gastric cancer, COVID-19, therapeutic drug

## Abstract

**Background:**

Coronavirus disease 2019 (COVID-19), an infectious disease caused by severe acute respiratory syndrome coronavirus-2 (SARS-CoV-2), has caused a global pandemic. Gastric cancer (GC) poses a great threat to people’s health, which is a high-risk factor for COVID-19. Previous studies have found some associations between GC and COVID-19, whereas the underlying molecular mechanisms are not well understood.

**Methods:**

We employed bioinformatics and systems biology to explore these links between GC and COVID-19. Gene expression profiles of COVID-19 (GSE196822) and GC (GSE179252) were obtained from the Gene Expression Omnibus (GEO) database. After identifying the shared differentially expressed genes (DEGs) for GC and COVID-19, functional annotation, protein-protein interaction (PPI) network, hub genes, transcriptional regulatory networks and candidate drugs were analyzed.

**Results:**

We identified 209 shared DEGs between COVID-19 and GC. Functional analyses highlighted immune-related pathways as key players in both diseases. Ten hub genes (*CDK1*, *KIF20A*, *TPX2*, *UBE2C*, *HJURP*, *CENPA*, *PLK1*, *MKI67*, *IFI6*, *IFIT2*) were identified. The transcription factor/gene and miRNA/gene interaction networks identified 38 transcription factors (TFs) and 234 miRNAs. More importantly, we identified ten potential therapeutic agents, including ciclopirox, resveratrol, etoposide, methotrexate, trifluridine, enterolactone, troglitazone, calcitriol, dasatinib and deferoxamine, some of which have been reported to improve and treat GC and COVID-19.

**Conclusion:**

This research offer valuable insights into the molecular interplay between COVID-19 and GC, potentially guiding future therapeutic strategies.

## 1 Introduction

Coronavirus disease 2019 (COVID-19) has caused a global pandemic, posing a significant health challenge worldwide and resulting in the deaths of over 6 million people (1). As the pandemic progresses, new variants of the severe acute respiratory syndrome coronavirus 2 (SARS-CoV-2) have emerged, with the World Health Organization (WHO) identifying variants of concern, including Alpha, Beta, Gamma, Delta, and Omicron (2, 3). SARS-CoV-2 enters the body by interacting with the angiotensin-converting enzyme 2 (ACE2) receptor and replicates within the epithelium, subsequently infecting surrounding cells (4). ACE2, a critical component of the renin-angiotensin system, has been identified as a membrane receptor for SARS-CoV-2 (5). Additionally, SARS-CoV-2 also enters host cells with the primary or auxiliary help of host proteases transmembrane protease serine 2 (TMPRSS2), FURIN (6), glucose-regulating protein 78 (GRP78) receptor (7), dipeptidyl peptidase 4 (DPP4) (8), cluster of differentiation 147 (CD147) transmembrane protein (9), tyrosine-protein kinase receptor UFO (AXL) (10), phosphatidylinositol 3-phosphate 5-kinase (PIKfyve) (11), two pore channel subtype 2 (TPC2) (12) and cathepsin L (13). Infection by SARS-CoV-2 alters alveolar vascular permeability, leading to lung injuries like pulmonary edema and pulmonary ischemia (14). Beyond the lungs, SARS-CoV-2 can spread to the brain, heart, gastrointestinal tract, and other organs through the bloodstream, causing severe complications (15–19). Due to the frequent genome reorganization of SARS-CoV-2, COVID-19 is likely to evolve and become seasonal epidemics (20). Clinical and epidemiological data suggest that underlying conditions, such as cancer, hypertension, cardiovascular disease, and diabetes, increase susceptibility to SARS-CoV-2 infection and can lead to more severe outcomes, including lung damage and death (21). Therefore, understanding how to treat COVID-19 in individuals with underlying diseases, including cancer, is of great research and clinical significance.

Gastric cancer (GC) is a major global health concern and the fourth leading cause of cancer-related deaths worldwide (22). GC incidence shows a strong geographic pattern, with the highest rates in East Asia, some South American and Eastern European countries, and the lowest in Africa and North America. More than 70% of GC cases occur in developing countries. GC patients typically have a poor prognosis and low long-term survival rates (23). Evidence suggests that GC patients are more vulnerable to SARS-CoV-2 infection, with immunotherapy and radiotherapy within three months of a COVID-19 diagnosis being risk factors for death (24). The expression profile of the SARS-CoV-2 host receptor ACE2 protein in the human gastrointestinal tract revealed that ACE2 was detectable in the gastric pits, fundic glands, gastric chief cells (pepsinogen-secreting cells), parietal cells (gastric acid-secreting cells), and pyloric glands, suggesting that gastric tissue may be susceptible to SARS-CoV-2 infection (25). Moreover, the intensity of ACE2 staining is significantly higher in gastric tumor tissues compared to adjacent non-tumor tissues, indicating that GC patients may be at increased risk of SARS-CoV-2 infection (25). A Mendelian randomization study also suggests a causal relationship between SARS-CoV-2 infection and an increased risk of gastric cancer (26). These findings underscore the importance of understanding the molecular mechanisms underlying the interaction between COVID-19 and gastric cancer.

In this study, we hypothesize that there are shared molecular mechanisms between COVID-19 and GC that could inform new therapeutic strategies. Specifically, we address the following research questions: 1. What are the common differentially expressed genes (DEGs) between COVID-19 and gastric cancer? 2. How do these shared DEGs contribute to the onset and progression of both diseases? 3. Can hub genes within the protein-protein interaction (PPI) network reveal key molecular players that may serve as potential therapeutic targets? 4. What are the potential drugs that could target these shared molecular mechanisms, and how might they contribute to the treatment of both COVID-19 and gastric cancer? To answer these questions, transcriptome profiles were obtained from the National Center for Biological Information (NCBI)-Gene Expression Omnibus (GEO) database. The datasets of COVID-19 and GC were studied to find DEGs for both diseases. These sets of DEGs were then compared to gain mutual DEGs. Moreover, the biological function of the common DEGs was analyzed to gain insights into its impact on disease onset and progression. A protein-protein interaction network was used to identify hub genes with the most obvious interactions and narrow down potential biomolecules. Next, the hub genes were used to establish the gene-regulatory network, predict potential drugs, and complete the gene-disease association network. This study identified the relationship between gastric cancer and COVID-19, potentially providing new ideas to assist in the treatment of GC and COVID-19.

## 2 Materials and methods

### 2.1 Data source and work flow

**FIGURE 1:**
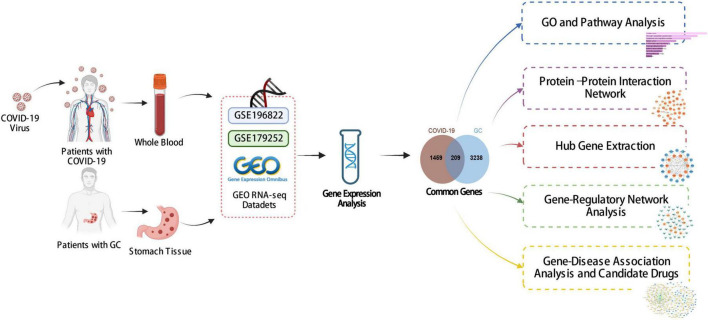
The overall work of this study.

Figure 1 shows the successive workflow of this study. To determine the mutual genetic interrelationship between SARS-CoV-2 infection and gastric cancer, we used the GEO database^1^ of NCBI to obtain RNA-seq datasets. For SARS-CoV-2 patients, we used GSE196822 dataset (27), which includes whole-blood transcriptome profiling of 34 COVID-19 patients and 9 healthy controls. The data came from high-throughput sequencing using the Illumina HiSeq 4000 (Homo sapiens). Gastric cancer (GSE179252) (28) consists of 38 gastric tumors and paired normal 38 gastric tissues which was based on Illumina HiSeq 4000 (Homo sapiens).

### 2.2 Identification of DEGs and common DEGs between COVID-19 and gastric cancer

The key target of the analysis is to find the DEGs for the datasets GSE196822 and GSE179252. The DEseq2 package (29) of R software (version 4.2.1) was used to identify the DEGs with false-discovery rate (FDR) < 0.05 and | log_2_ Fold Change| > 1. To extract the shared DEGs between COVID-19 and gastric cancer, an online VENN visual tool called Jvenn^2^ was used (30).

### 2.3 Gene ontology and pathway enrichment analysis

The use of gene ontology (GO) enrichment methods is widespread for demonstrating the relationship between genes and GO terms, and the GO database is a comprehensive resource on gene and gene product functions (31). GO annotated sources for biological process (BP), molecular function (MF), and cellular component (CC) were retrieved from the GO database (32). To identify pathways shared by GC and COVID-19, we considered the following three repositories as the origin of pathway classification: WikiPathways, Reactome, and the Kyoto Encyclopedia of Genes and Genomes (KEGG). We used Enrichr (33)^3^ for gene ontology and pathway enrichment investigations. To quantify the top pathways and functional items, a standardized index with a *P*-value < 0.05 was utilized.

### 2.4 Protein–protein interaction network analysis and hub genes extraction

STRING (version 11.5) database,^4^ an online protein-protein association networks platform, has been used to construct the PPI network with a filter condition (combined score > 0.5) (34). All the common DEGs between COVID-19 and GC were used to build the PPI network. Then, the PPI network was consumed into Cytoscape (v.3.9.1) for visual representation and hub genes’ recognition (35). We used Maximal Clique Centrality (MCC) method of Cytohubba (a plugin of Cytoscape)^5^ to identify the top 10 hub genes from the PPI network (36). At the same time, Cytohubba’s proximity ranking characteristics helped us to identify the shortest reachable pathways linking hub genes.

### 2.5 Identification of miRNAs–gene and transcription factors–gene interactions

Transcription factors (TFs) are proteins attached to specific genes that control the genetic information’s transcription rate; as such, they are essential for molecular insight (37). Our approach involved utilizing the NetworkAnalyst platform (version 3.0) (38) to identify topologically feasible TFs from the JASPAR database that could be potentially integrated with hub genes. JASPAR is a publicly accessible database that compiles information on TFs across six taxonomic groups for various species (39). miRNAs targeting gene interactions are also included in studies to identify miRNAs that tend to bind to gene transcripts and thus negatively impact protein production. We used Network-Analyst to analyze miRNAs-gene interactions from Tarbase (version 8.0) (40) databases.

### 2.6 Identification of drug candidates

Another emphasis of this study was to use the hub genes of COVID-19 and GC to predict protein-drug interactions (PDIs) or drug molecule recognition. Using Enrichr’s disease/drug functions, based on hub genes, drug molecules were predicted from the Drug Signatures Database (DSigDB)^6^ (41), which contains 17,389 unique chemicals that span 19,531 genes and has 22,527 gene sets.

### 2.7 Gene-disease association analysis

DisGeNET^7^ (42) is a platform that integrates and standardizes data on genes associated with diseases from diverse sources. Currently, DisGeNET has information on about 24,000 illnesses and features, 17,000 genes, and 117,000 genetic variations. To identify the relationship between relevant diseases and common DEGs, we use DisGeNET, Network-Analyst and Cytoscape to investigate the relationship between genes and diseases.

## 3 Results

### 3.1 Determination of DEGs and common DEGs of GC and COVID-19

**FIGURE 2:**
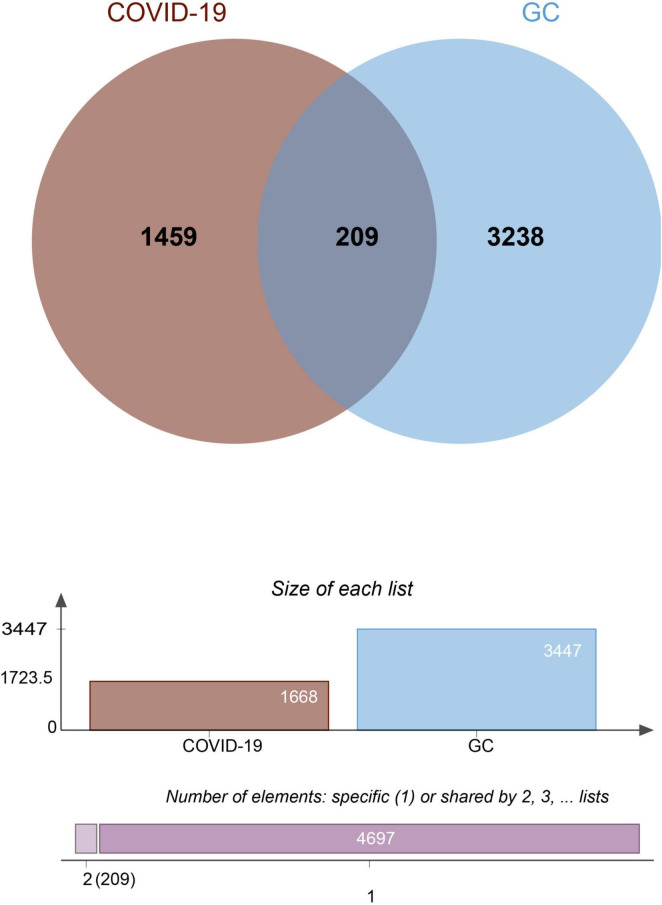
The Venn diagram showed 209 shared DEGs between COVID-19 and GC.

To investigate the correlation and influence between GC and COVID-19, we analyzed human RNA-seq datasets from GEO and identified shared DEGs that may trigger both COVID-19 and GC. In this study, 1,668 genes were found to be differentially expressed in COVID-19, including 839 up regulated DEGs and 829 down regulated DEGs (Supplementary Table 1). Similarly, 3,447 DEGs were identified in the GC data, including 1045 up regulated DEGs and 2402 down regulated DEGs (Supplementary Table 2). The information about the two datasets has been integrated in Table 1. To find shared DEGs between GC and COVID-19, we performed a cross-comparative evaluation using Jvenn and identified 209 common DEGs in both datasets (Figure 2 and Supplementary Table 3). There are multiple genes in common between GC and COVID-19, suggesting some similarity between the two diseases.

**TABLE 1:** Overview of datasets with their geo-features and their quantitative measurements in this analysis.

Disease name	GEO accession	GEO platform	Total DEGs count	Upregulated DEGs count	Downregulated DEGs count
COVID-19	GSE196822	GPL20301	1,668	839	829
Gastric cancer	GSE179252	GPL20301	3,447	1,045	2,402

### 3.2 Analyses of GO and pathway enrichment

**FIGURE 3:**
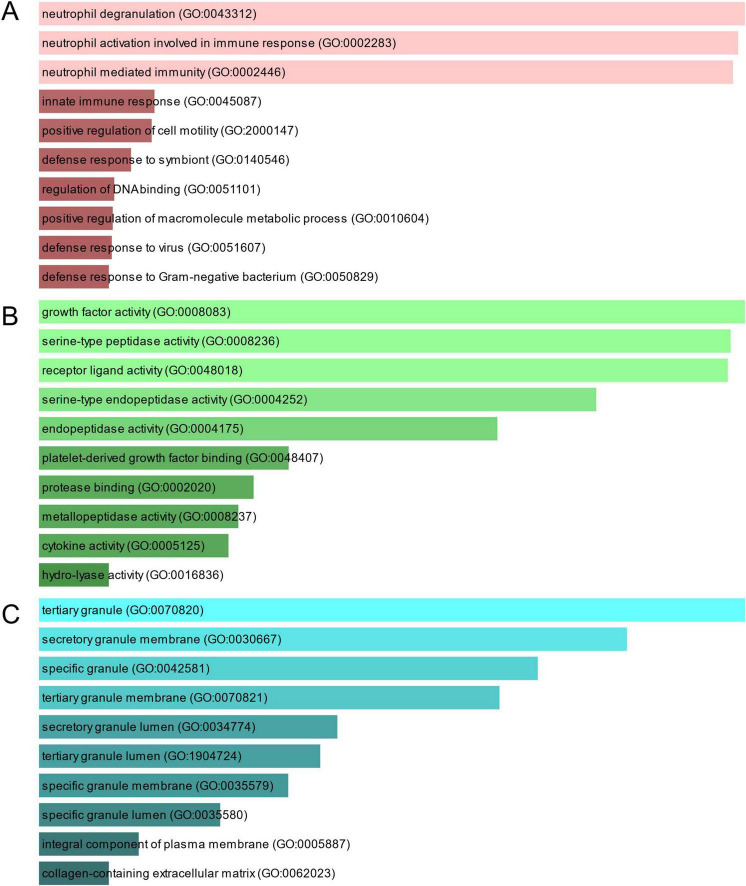
The bar graphs depicting the gene ontology enrichment analysis of shared DEGs between COVID-19 and gastric cancer. **(A)** biological process. **(B)** molecular function. **(C)** cellular component. The lighter the color, the more significant it is.

To investigate the enrichment pathways and biological significance of common DEGs between COVID-19 and GC, we used Enrichr for gene functional annotation. Figure 3 and Table 2 summarized the top 10 enriched GO categories in the biological process, molecular function and cellular component categories. Notably, common DEGs were significantly enriched in immune-related pathways, which include neutrophil degranulation (GO:0043312), neutrophil activation involved in immune response (GO:0002283), neutrophil mediated immunity (GO:0002446), innate immune response (GO:0045087), defense response to virus (GO:0051607), receptor ligand activity (GO:0048018), and cytokine activity (GO:0005125).

**TABLE 2:** Ontological analysis of common DEGs between SARS-CoV-2 and GC.

Category	GO ID	Term	*P*-value	Genes
GO biological process	GO:0043312	Neutrophil degranulation	4.42E-14	CDA; CRISP3; GPR84; SLC2A5; ABCA13; TRPM2; PLAU; ANPEP; CLEC5A; OLR1; CD177; MGAM; SLC11A1; HSPA6; MCEMP1; AZU1; RNASE2; LILRB3; OLFM4; MMP9; OSCAR; CHIT1; CEACAM6; CYSTM1; BPI; S100P; HSPA1A; LTF
	GO:0002283	Neutrophil activation involved in immune response	5.43E-14	CDA; CRISP3; GPR84; SLC2A5; ABCA13; TRPM2; PLAU; ANPEP; CLEC5A; OLR1; CD177; MGAM; SLC11A1; HSPA6; MCEMP1; AZU1; RNASE2; LILRB3; OLFM4; MMP9; OSCAR; CHIT1; CEACAM6; CYSTM1; BPI; S100P; HSPA1A; LTF
	GO:0002446	Neutrophil mediated immunity	6.33E-14	CDA; CRISP3; GPR84; SLC2A5; ABCA13; TRPM2; PLAU; ANPEP; CLEC5A; OLR1; CD177; MGAM; SLC11A1; HSPA6; MCEMP1; AZU1; RNASE2; LILRB3; OLFM4; MMP9; OSCAR; CHIT1; CEACAM6; CYSTM1; BPI; S100P; HSPA1A; LTF
	GO:0045087	Innate immune response	2.01E-06	IFITM3; IFITM1; SLC11A1; F12; CRISP3; IFI6; ISG15; RNASE2; CXCL16; TREML1; CLEC5A; BPI; CLEC4E; LTF
	GO:2000147	Positive regulation of cell motility	2.19E-06	COL1A1; SEMA6B; CEACAM6; PLAU; MDK; EGF; HGF; GPER1; LEF1; EPHB2; MMP9; CXCL16
	GO:0140546	Defense response to symbiont	4.05E-06	IFITM3; IFITM1; IFI6; ISG15; AZU1; RNASE2; RNASE1; IFI44L; IFIT2
	GO:0051101	Regulation of DNA binding	6.68E-06	CDT1; EGF; LEF1; HJURP; E2F1; MMP9
	GO:0010604	Positive regulation of macromolecule metabolic process	7.00E-06	SLC24A3; EGF; SLC11A1; HPN; LEF1; PCSK9; AZU1; INHBA; LGALS9C; GPER1; TMEM119; E2F1; EPHB2; FGFR4; HSPA1A
	GO:0051607	Defense response to virus	7.20E-06	IFITM3; IFITM1; IFI6; ISG15; AZU1; RNASE2; RNASE1; IFI44L; IFIT2
	GO:0050829	Defense response to Gram-negative bacterium	7.86E-06	SELP; SLC11A1; SERPINE1; BPI; AZU1; RNASE2; LTF
GO molecular function	GO:0008083	Growth factor activity	2.26E-04	MDK; EGF; HGF; CLEC11A; OSM; INHBA
	GO:0008236	Serine-type peptidase activity	2.50E-04	PLAU; HGF; HTRA3; F12; HPN; PCSK9; MMP9
	GO:0048018	Receptor ligand activity	2.54E-04	SEMA6B; GDF15; MDK; EGF; IL34; HGF; CLEC11A; OSM; TIMP1; INHBA; ERFE
	GO:0004252	Serine-type endopeptidase activity	6.23E-04	PLAU; HGF; F12; HPN; PCSK9; MMP9
	GO:0004175	Endopeptidase activity	0.001221	ADAM28; ADAMTS2; PLAU; F12; HGF; HTRA3; HPN; PCSK9; MMP9; TRABD2A
	GO:0048407	Platelet-derived growth factor binding	0.005057	COL1A1; COL1A2
	GO:0002020	Protease binding	0.006418	COL1A1; COL1A2; SERPINE1; TIMP1; CD177
	GO:0008237	Metallopeptidase activity	0.007122	ADAMTS2; ADAM28; ANPEP; MMP9; TRABD2A
	GO:0005125	Cytokine activity	0.007615	GDF15; IL34; OSM; INHBA; TIMP1; CXCL16
	GO:0016836	Hydro-lyase activity	0.017196	CBS; CA4; ECHDC3
GO cellular component	GO:0070820	Tertiary granule	6.32E-13	CDA; MGAM; SLC11A1; CRISP3; MCEMP1; GPR84; OLFM4; MMP9; OSCAR; CHIT1; TRPM2; PLAU; CLEC5A; OLR1; CYSTM1; CD177; LTF
	GO:0030667	Secretory granule membrane	3.46E-11	MGAM; SLC11A1; CCDC136; MCEMP1; AZU1; GPR84; LILRB3; SLC2A5; ABCA13; SELP; TRPM2; CEACAM6; PLAU; ANPEP; CLEC5A; CA4; OLR1; CYSTM1; CD177
	GO:0042581	Specific granule	7.11E-10	CRISP3; MCEMP1; GPR84; OLFM4; SLC2A5; OSCAR; CHIT1; TRPM2; PLAU; CLEC5A; OLR1; BPI; CD177; LTF
	GO:0070821	Tertiary granule membrane	2.60E-09	TRPM2; MGAM; PLAU; SLC11A1; CLEC5A; OLR1; MCEMP1; CYSTM1; GPR84; CD177
	GO:0034774	Secretory granule lumen	6.37E-07	CDA; EGF; HGF; CRISP3; SERPINE1; HSPA6; AZU1; RNASE2; OLFM4; OSCAR; CHIT1; BPI; S100P; TIMP1; LTF
	GO:1904724	Tertiary granule lumen	1.14E-06	CHIT1; CDA; CRISP3; OLFM4; MMP9; OSCAR; LTF
	GO:0035579	Specific granule membrane	3.37E-06	TRPM2; PLAU; CLEC5A; OLR1; MCEMP1; GPR84; SLC2A5; CD177
	GO:0035580	Specific granule lumen	3.38E-05	CHIT1; CRISP3; BPI; OLFM4; OSCAR; LTF
	GO:0005887	Integral component of plasma membrane	5.36E-04	KCNG2; CNTNAP1; CSF3R; SLC24A3; PTGDR2; HPN; SLC1A3; GPR84; SLC2A5; TRPM2; HRH2; GPER1; CLEC5A; OLR1; EPHB2; FCGR1A; NTSR1; PLXNA4; SEMA6B; SLC11A1; LILRB3; SELP; SLCO4A1; FGFR4; F2RL2; GPR19; SLC28A3; TRABD2A
	GO:0062023	Collagen-containing extracellular matrix	0.001478	COL1A1; COL1A2; GDF15; MDK; F12; COL7A1; SERPINE1; COL9A2; MMP9; LOXL1; TGM2

**FIGURE 4:**
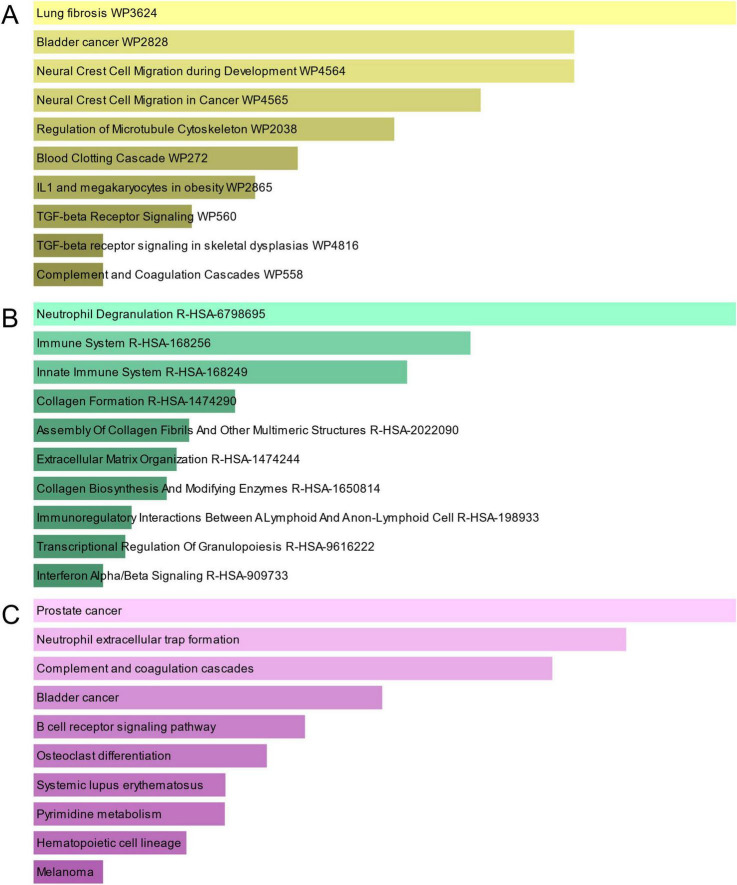
The bar graphs of pathway enrichment analysis of mutual DEGs between COVID-19 and GC. **(A)** WikiPathway. **(B)** Reactome pathway. **(C)** KEGG. The lighter the color, the more significant it is.

Pathway analysis can highlight how underlying molecular and biological processes interact (43). Figure 4 and Table 3 show the main pathways of common DEGs enrichment in WikiPathways, Reactome, and KEGG. Pathway enrichment analysis showed that common DEGs are mainly involved in the regulation of immune-related pathways, including TGF-beta Receptor Signaling WP560, Neutrophil Degranulation R-HSA-6798695, Immune System R-HSA-168256, Innate Immune System R-HSA-168249, Immunoregulatory Interactions Between A Lymphoid And A non-Lymphoid Cell R-HSA-198933, Transcriptional Regulation Of Granulopoiesis R-HSA-9616222, Interferon Alpha/Beta Signaling R-HSA-909733 and B cell receptor signaling pathway. These results provide strong evidence that these common DEGs play a role in the onset and development of COVID-19 and GC through immune-related pathways.

**TABLE 3:** Pathway enrichment analysis of common DEGs between SARS-CoV-2 and GC.

Category	Term	*P*-value	Genes
WikiPathways	Lung fibrosis WP3624	3.99E-04	PLAU; EGF; HGF; TIMP1; MMP9
	Bladder cancer WP2828	6.44E-04	EGF; E2F1; MMP9; TYMP
	Neural crest cell migration during development WP4564	6.44E-04	AKT3; EPHB2; MMP9; F2RL2
	Neural crest cell migration in cancer WP4565	8.50E-04	AKT3; EPHB2; MMP9; F2RL2
	Regulation of microtubule cytoskeleton WP2038	0.001099	CFL2; CDK1; EPHB2; F2RL2
	Blood clotting cascade WP272	0.001462	PLAU; F12; SERPINE1
	IL1 and megakaryocytes in obesity WP2865	0.001659	TIMP1; PLA2G7; MMP9
	TGF-beta receptor signaling WP560	0.002002	EGF; SERPINE1; LEF1; INHBA
	TGF-beta receptor signaling in skeletal dysplasias WP4816	0.002605	EGF; SERPINE1; LEF1; INHBA
Reactome	Neutrophil degranulation R-HSA-6798695	2.96E-15	LILRA6; CDA; CRISP3; GPR84; SLC2A5; ABCA13; TRPM2; PLAU; ANPEP; CLEC5A; OLR1; CD177; MGAM; SLC11A1; HSPA6; MCEMP1; AZU1; RNASE2; OLFM4; RNASE1; MMP9; OSCAR; CHIT1; CEACAM6; CYSTM1; BPI; S100P; HSPA1A; LTF
	Immune system R-HSA-168256	1.79E-10	IFITM3; CDA; IFITM1; CSF3R; CRISP3; SLC2A5; ABCA13; IFIT2; PLAU; ANPEP; AKT3; CLEC5A; OLR1; TIMP1; CD177; TNFRSF12A; IL1R2; SLC11A1; MCEMP1; RNASE2; OLFM4; RNASE1; MMP9; OSCAR; CHIT1; CEACAM6; BPI; KIF20A; CLEC4E; LTF; LILRA6; IFI6; GPR84; LILRA5; TRPM2; FCGR1A; MGAM; UBE2C; SIGLEC11; IL34; HSPA6; ISG15; AZU1; FBXO32; TREML1; COL1A2; CYSTM1; S100P; GSDME; HSPA1A
	Innate immune system R-HSA-168249	2.48E-09	LILRA6; CDA; CRISP3; GPR84; SLC2A5; ABCA13; TRPM2; PLAU; ANPEP; CLEC5A; OLR1; FCGR1A; CD177; MGAM; SLC11A1; HSPA6; MCEMP1; ISG15; AZU1; RNASE2; OLFM4; RNASE1; MMP9; OSCAR; CHIT1; CEACAM6; CYSTM1; BPI; S100P; CLEC4E; GSDME; HSPA1A; LTF
	Collagen formation R-HSA-1474290	3.10E-06	COL1A1; ADAMTS2; COL1A2; PCOLCE2; COL7A1; COL9A2; MMP9; LOXL1
	Assembly of collagen fibrils and other multimeric structures R-HSA-2022090	2.08E-05	COL1A1; COL1A2; COL7A1; COL9A2; MMP9; LOXL1
	Extracellular matrix organization R-HSA-1474244	3.50E-05	COL1A1; CAPN13; ADAMTS2; COL1A2; PCOLCE2; CEACAM6; COL7A1; SERPINE1; COL9A2; TIMP1; MMP9; LOXL1
	Collagen biosynthesis and modifying enzymes R-HSA-1650814	5.27E-05	COL1A1; ADAMTS2; COL1A2; PCOLCE2; COL7A1; COL9A2
	Immunoregulatory interactions between a lymphoid and a non-lymphoid cell R-HSA-198933	2.26E-04	TREML1; LILRA6; IFITM1; SIGLEC11; FCGR1A; OSCAR; LILRA5
	Transcriptional regulation of granulopoiesis R-HSA-9616222	2.93E-04	H2BC9; CSF3R; LEF1; E2F1; H2BC17
	Interferon alpha/beta signaling R-HSA-909733	7.39E-04	IFITM3; IFITM1; IFI6; ISG15; IFIT2
KEGG	Prostate cancer	5.07E-05	PLAU; EGF; IL1R2; AKT3; LEF1; E2F1; MMP9
	Neutrophil extracellular trap formation	1.15E-04	H4C8; SELP; H2BC9; H2BC7; CR1L; AKT3; AZU1; FCGR1A; H2BC17
	Complement and coagulation cascades	1.99E-04	PLAU; CR1L; F12; SERPINE1; F2RL2; C2
	Bladder cancer	7.09E-04	EGF; E2F1; MMP9; TYMP
	B cell receptor signaling pathway	0.00126	LILRA6; IFITM1; AKT3; LILRB3; LILRA5
	Osteoclast differentiation	0.001674	LILRA6; AKT3; FCGR1A; LILRB3; OSCAR; LILRA5
	Systemic lupus erythematosus	0.00228	H4C8; H2BC9; H2BC7; FCGR1A; H2BC17; C2
	Pyrimidine metabolism	0.00229	CDA; ENPP3; UPP1; TYMP
	Hematopoietic cell lineage	0.003049	CSF3R; CR1L; ANPEP; IL1R2; FCGR1A
	Melanoma	0.005675	EGF; HGF; AKT3; E2F1

### 3.3 Building PPI network and selecting of hub genes

**FIGURE 5:**
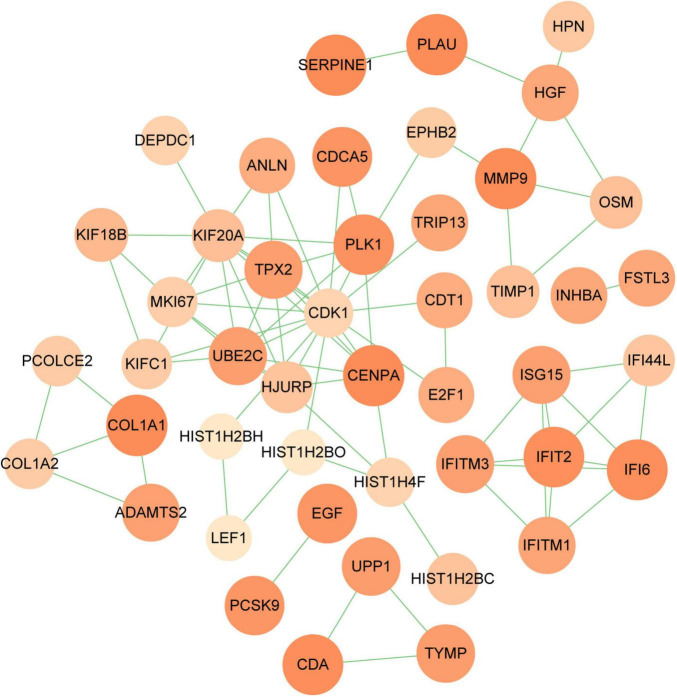
PPI network of shared DEGs between COVID-19 and GC. The DEGs in the figure are represented by circles and the edges mean the connections between the nodes. The PPI network consists of 46 nodes and 82 edges.

**FIGURE 6:**
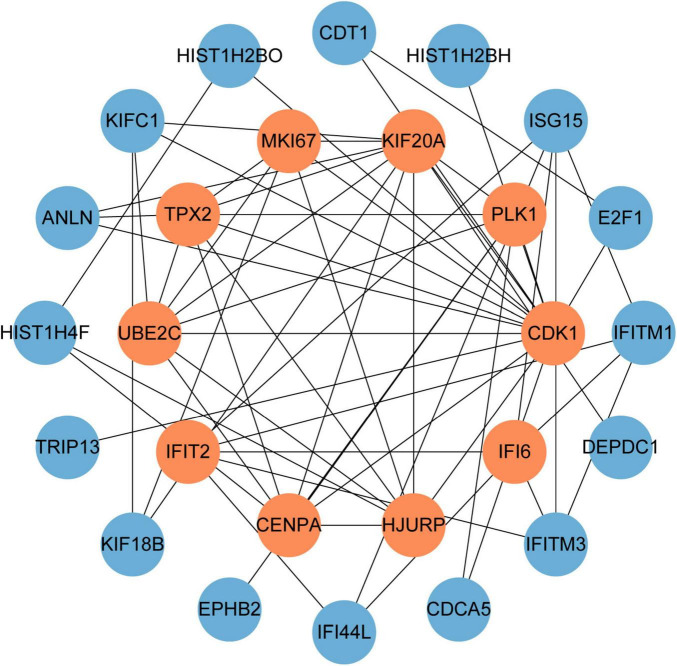
The hub genes were obtained from the PPI network. The nodes in orange represent the top 10 prominent hub genes and the interactions among them and other molecules. This network contains 26 nodes and 60 edges.

PPI networks can visualize the interrelationships between different proteins and can help us understand the underlying mechanisms by which proteins interact (44). Using STRING and Cytoscape, we built and visualized a PPI network of shared DEGs between COVID-19 and GC, which encompasses 46 nodes and 82 edges, as depicted in Figure 5. The most entangled nodes among them are hub genes. These hub genes have the potential to serve as biomarkers and may offer novel insights into therapeutic approaches. The top 10 hub genes with the highest MCC scores were identified using the cytoHubba plugin of Cytoscape, namely *CDK1*, *KIF20A*, *TPX2*, *UBE2C*, *HJURP*, *CENPA*, *PLK1*, *MKI67*, *IFI6*, and *IFIT2* (Figure 6 and Supplementary Table 4).

### 3.4 Identifying of transcription regulatory network

**FIGURE 7:**
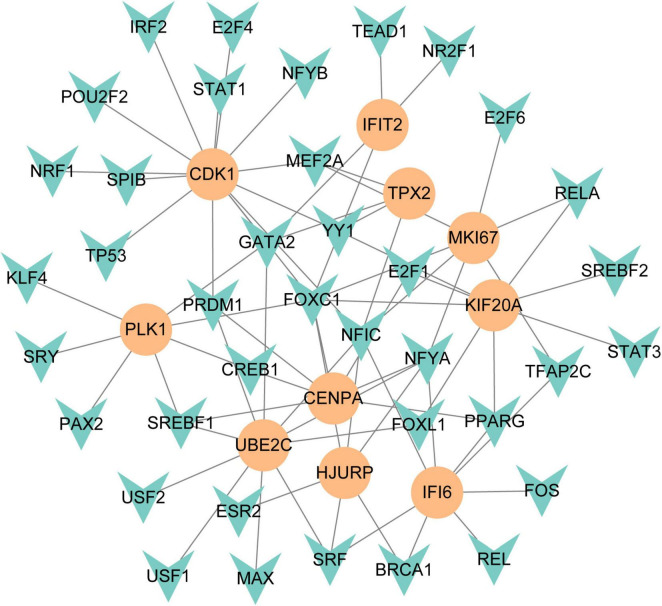
The regulatory network of TFs-gene. The circles represent hub gene, and transcription factor was represented by quadrilateral shape. The network contains 38 nodes and 75 edges.

**FIGURE 8:**
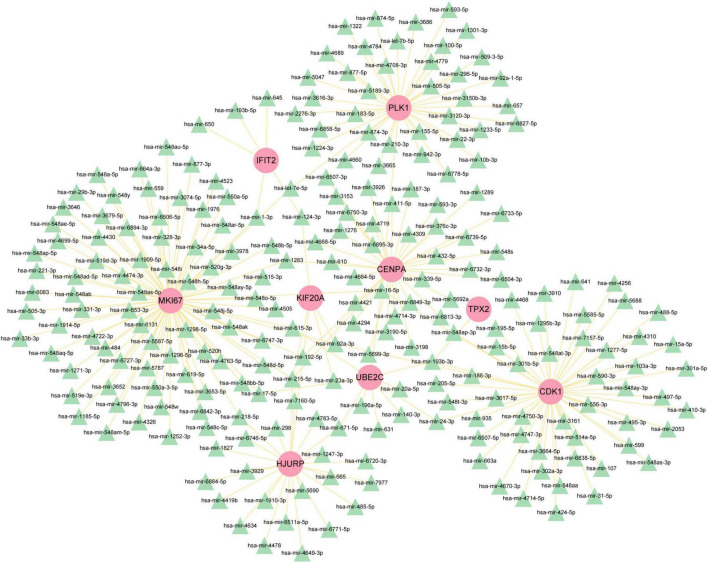
The regulatory network of miRNAs-gene. The pink circle represents the hub gene and the green triangle represents the miRNA. The network contains 243 nodes and 256 edges.

To figure out how hub genes modulate COVID-19 and GC at the transcriptional level, this study also investigated the interaction between TFs and genes, as well as miRNAs. In this study, a network-based approach was used to decode regulatory transcription factors and miRNAs as a way to gain insight into hub genes and find substantial changes that occur at the transcriptional level. As shown in Figure 7, the figure exhibits the interaction network of 38 transcription factors, such as ESR2, REL, SRY, SPIB, NR2F1, BRCA1, FOS, CREB1, FOXC1 and GATA2 (Supplementary Table 5). Similarly, Figure 8 represents the interaction network of miRNAs regulators and hub genes, containing 234 miRNAs, such as hsa-mir-16-5p, hsa-mir-192-5p, hsa-mir-215-5p, hsa-mir-92a-3p, hsa-mir-193b-3p, hsa-let-7e-5p, hsa-mir-1283, hsa-mir-218-5p, hsa-mir-1-3p and hsa-mir-671-5p (Supplementary Table 6).

### 3.5 Determination of candidate drugs

To search for potential drugs to treat COVID-19 and GC, possible drug molecules were predicted based on the transcriptional characteristics from the DSigDB database (45). The top 8 compounds were identified according to their *P*-values (Table 4). The potential drug compounds were ciclopirox, resveratrol, etoposide, methotrexate, trifluridine, enterolactone, troglitazone, calcitriol, dasatinib and deferoxamine. These drugs have the possibility to be used as treatment for GC and COVID-19.

**TABLE 4:** Drug candidates combined with hub genes.

Name	*P*-value	Molecular formula	Structure
Ciclopirox	4.08E-16	C_12_H_17_NO_2_	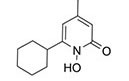
Resveratrol	9.92E-15	C_14_H_12_O_3_	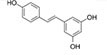
Etoposide	2.88E-14	C_29_H_32_O_13_	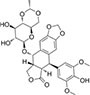
Methotrexate	4.77E-14	C_20_H_22_N_8_O_5_	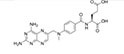
Trifluridine	2.23E-12	C_10_H_11_F_3_N_2_O_5_	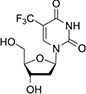
Enterolactone	1.24E-09	C_18_H_18_O_4_	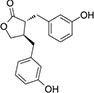
Troglitazone	4.13E-09	C_24_H_27_NO_5_S	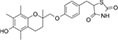
Calcitriol	7.41E-09	C_27_H_44_O_3_	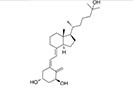
Dasatinib	4.68E-08	C_22_H_26_ClN_7_O_2_S	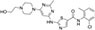
Deferoxamine	1.02E-07	C_25_H_48_N_6_O_8_	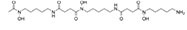

### 3.6 Exploration of gene-disease associations

**FIGURE 9:**
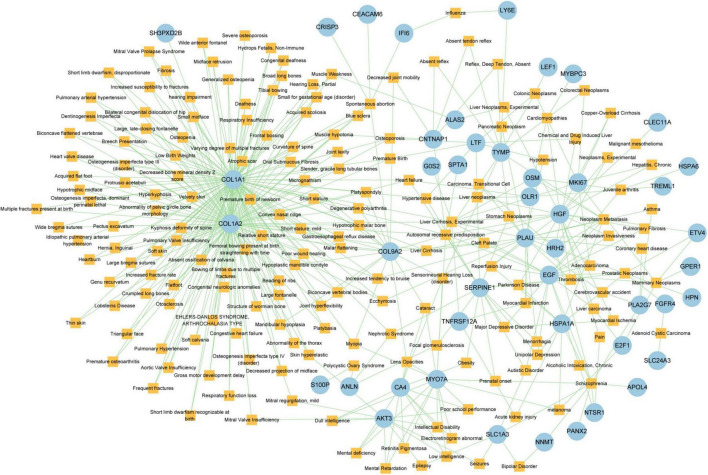
The Gene-disease association network. The square nodes represent diseases and the round nodes represent DEGs.

Different diseases can generally be considered to be associated with each other if they have one or more similar genes. With the DisGeNET database, Network-Analyst was used to analyze gene-disease associations (Figure 9). Network-Analyst further revealed the stomach neoplasms, colonic neoplasms, autosomal recessive predisposition, liver cirrhosis experimental, mammary neoplasms, neoplasm metastasis, and prostatic neoplasms to be most associated with the identified COVID-19/GC-related common DEGs.

## 4 Discussion

A strong correlation between COVID-19 and GC has been reported (24–26), with GC patients being more susceptible to viral infection post-surgery, chemotherapy, or radiotherapy. Once infected, the disease progression in GC patients is often more rapid and severe, leading to higher mortality rates. This study aims to explore the molecular mechanisms underlying this correlation to potentially inform new therapeutic strategies.

Here, we identified 209 common DEGs between COVID-19 and GC and explored the biological function of shared DEGs in the pathogenesis of COVID-19 and GC. Notably, these common DEGs are significantly enriched in many immune-related pathways. Alterations in neutrophil number and function have been identified as one of the immunopathological markers associated with severe COVID-19 (46). Studies have shown that neutrophils in healthy individuals can become dysfunctional in degranulation due to factors secreted by epithelial cells which were infected by SARS-CoV-2 (47). Also, neutrophils play an important role in tumor progression and metastasis (48). Cytokine activity was affected by SARS-CoV-2 infection. Impaired acquired immune responses and uncontrolled inflammatory innate responses to SARS-CoV-2 may lead to cytokine storms (49). The occurrence and development of tumors are actually closely related to the immune system, and cancer patients generally have immune dysfunction and low resistance. At the same time, in the process of tumor treatment, including surgery, chemotherapy and radiotherapy, it has a great impact on the body, which will have a certain impact on the patient’s immune system. Targeting immune-related pathways offers a promising avenue for the development of therapeutic strategies against both COVID-19 and gastric cancer.

The common DEGs are utilized to construct the PPI network, in which the hub gene is the most significant regulator in the common pathogenetic processes of GC and COVID-19. CDK1 is an important regulator of cell cycle at G1/S and G2/M checkpoints (50). It has been reported that CDK1 is highly expressed in gastric cancer. Phosphorylation of islet-1 serine 269 by CDK1 can increase its transcriptional activity and promote the proliferation of gastric cancer cells (51), and inhibition of CDK1 can inhibit the proliferation, migration and invasion of GC cells (52). CDK1 is highly expressed in PBMCs of COVID-19 patients and is involved in the apoptosis process; CDK1 may also be associated with a worsening of the course of COVID-19, which is characterized by an extreme decrease in immune cells (53). KIF20A (also known as mitotic kinesin-like protein 2, MKlp2) transports chromosomes during mitosis and plays a key role in cell division. KIF20A is highly expressed in almost all cancers, including gastric cancer (54), melanoma (55), hepatocellular carcinoma (56), and breast cancer (57). Several studies have also shown that KIF20A is a hub gene involved in SARS-CoV-2 infection (58, 59). TPX2 is a microtubule-associated protein that activates the cell cycle kinase protein Aurora-A, which then plays an vital role in spindle formation in mitosis (60), and high TPX2 expression is associated with tumor progression and low survival in gastric cancer (61). TPX2 may be a novel COVID-19 intervention target and biomarker (62). Overexpression of UBE2C is associated with poor prognosis of patients with gastric cancer, and it is also a potential biomarker for intestinal-type gastric cancer (63). A study of peripheral blood transcriptome sequencing in patients with pneumonia found that the expression of UBE2C in patients with severe pneumonia was higher than that in patients with mild pneumonia (64). HJURP (65), CENPA (66), PLK1 (67) and IFI6 (68) were found to significantly increase in gastric cancer tissues compared with normal tissues. In addition, HJURP (69), PLK1 (58), MKI67 (70) and IFI6 (71) have been identified as potential therapeutic target for COVID-19 patients. IFIT2 have been proved to have important roles in regulating apoptosis. Chen et al. (72) showed that decreased expression of IFIT2 promotes gastric cancer progression and predicts poor patient prognosis. Similarly, the significant downregulation of IFIT2 has been observed in patients with severe COVID-19 (73). These findings suggest that targeting these hub genes could be a promising therapeutic strategy in managing both diseases.

In this study, we identified a variety of compounds and medications that may treat COVID-19 and GC, including ciclopirox, resveratrol, etoposide, methotrexate, trifluridine, enterolactone, troglitazone, calcitriol, dasatinib and deferoxamine. Ciclopirox is an antifungal drug that was recently identified as a promising cancer treatment (74). Ciclopirox regulates the growth and autophagic cell death of GC cells by regulating the phosphorylation of STAT3 at Tyr705 and Ser727 residues, and suggests that ciclopirox may be a potential treatment for GC (75). Consistent with this study, Zhang et al. (76) identified ciclopirox as a potential therapeutic agent for the treatment of patients with SARS-CoV-2 infection through drug prediction and simulated docking patterns. Resveratrol is considered an anti-inflammatory and antiviral agent. Resveratrol inhibits the progression of gastric cancer by anti-inflammatory, antioxidant (77), antibacterial (78), inducing cell cycle arrest (79), promoting apoptosis (80), and inhibiting proliferation (81). Besides, resveratrol downregulates neutrophil extracellular traps (NETs) generation by neutrophils in patients with severe COVID-19 (82). Similarly, network pharmacology has shown that resveratrol can alleviate COVID-19-related hyperinflammation (83). Etoposide is a class of anticancer drugs (84). Etoposide induces cell death through the mitochondria-dependent effects of p53 (85). The interaction of etoposide with pertuzumab or trastuzumab induces programmed cell death in gastric cancer cells through exogenous and endogenous apoptotic pathways (86). Besides, etoposide may be a very effective treatment to protect critically ill patients from death caused by a storm of COVID-19-specific cytokines (87). Methotrexate is a tightly bound dihydrofolate reductase (DHFR) inhibitor that is used both as an antineoplastic agent and as an immunosuppressant (88). Clinical and experimental data suggest that methotrexate has a protective effect on SARS-CoV-2 infection by downregulating ACE2 (89). Trifluridine/tipiracil could be a new treatment option for patients with heavily pretreated advanced gastric cancer after progression on, or intolerance to, two or more previous lines of chemotherapy, including a fluoropyrimidine, a platinum agent, a taxane or irinotecan (or both), and an anti-HER2 therapy (in patients with HER2-positive disease) (90). Similarly, trifluridine is considered to have good anti-SARS-CoV-2 capability by virtual screening, ADME/T, and binding free energy analysis (91, 92). Enterolactone is a bioactive phenolic metabolite known as mammalian lignans derived from dietary lignans (93). Enterolactone has potent anti-cancer and/or protective properties against different cancers, including gastric (94), breast (95), colorectal (96), lung (97), ovarian, endometrial (98), and hepatocellular carcinoma (99). In another bioinformatics and systems biology analysis, enterolactone was similarly identified as a potential treatment for COVID-19 (70). Troglitazone induces apoptosis in gastric cancer cells through the NAG-1 pathway (100). In addition, troglitazone has been identified as a potential inhibitor of SARS-CoV-2 replicase (101). Calcitriol alleviates COVID-19 complications by modulating pro-inflammatory cytokines, antiviral proteins, and autophagy (102). Similarly, there was an improvement in peripheral arterial oxygen saturation and inspired oxygen fraction in hospitalized patients with COVID-19 treated with calcitriol (103). Dasatinib promotes TRAIL-mediated apoptosis by upregulating CHOP-dependent death receptor 5 in gastric cancer (104). Dasatinib can reduce SARS-CoV-2-related mortality, delay its onset, and reduce the number of other clinical symptoms (105). Deferoxamine is a widely used iron chelator used to treat iron overload. Deferoxamine targets mitochondria and impair mitochondrial respiration and [Fe-S] cluster/heme biogenesis in cancer cells, thereby inhibiting tumor proliferation and migration and inducing cell death (106). Deferoxamine has iron chelation, antiviral, and immunomodulatory effects to help control SARS-CoV-2 (107).

This study explores the relationship between COVID-19 and GC using bioinformatics and systems biology approaches; however, several limitations should be acknowledged. First, our analysis relies on data retrieved from specific public databases, which may introduce biases related to sample selection, data collection methods, and population differences. Second, the gene expression data used in this study may be subject to methodological biases, including batch effects and variations in experimental conditions across datasets. Although we identified several hub genes and pathways potentially linking COVID-19 and GC, our study is based on computational predictions. Experimental validation through *in vitro* and *in vivo* studies is necessary to confirm the biological significance and therapeutic relevance of the identified targets. Additionally, translating these findings into clinical practice presents significant challenges, requiring further research to ensure these insights can be effectively applied in therapeutic settings.

## 5 Conclusion

In this study, transcriptome analysis was applied to summarize the relationship between gastric cancer and COVID-19. DEGs for GC and COVID-19 were obtained in the GEO dataset, 209 shared DEGs were identified, and associations between gastric cancer and COVID-19 were found. To clarify what role these DEGs play at the transcriptional level, enrichment analysis was conducted. We also used these common DEGs to obtain a PPI network and defined the 10 most important hub genes: *CDK1*, *KIF20A*, *TPX2*, *UBE2C*, *HJURP*, *CENPA*, *PLK1*, *MKI67*, *IFI6*, and *IFIT2*. Besides, we established a TF-gene and miRNA-gene interaction network for hub genes and identified key TFs and miRNAs. More importantly, we identified a variety of compounds and drugs that may treat COVID-19 and GC, such as ciclopirox, resveratrol, etoposide, methotrexate, trifluridine, enterolactone, troglitazone, calcitriol, dasatinib and deferoxamine. This study shows new possibilities for the treatment of COVID-19 and GC.

## Data Availability

The original contributions presented in this study are included in this article/Supplementary material, further inquiries can be directed to the corresponding authors.

## Footnotes

1
https://www.ncbi.nlm.nih.gov/geo/

2
https://jvenn.toulouse.inra.fr/app/example.html

3
https://maayanlab.cloud/Enrichr/

4
https://string-db.org/

5
http://apps.cytoscape.org/apps/cytohubba

6
http://tanlab.ucdenver.edu/DSigDB

7
http://www.disgenet.org/

## Supplementary material

Supplementary Table 1 The differentially expressed genes of COVID-19.

Supplementary Table 2 The differentially expressed genes of GC.

Supplementary Table 3 Common DEGs between COVID-19 and GC.

Supplementary Table 4 Hub genes in network string_interactions ranked by MCC method.

Supplementary Table 5 The TFs and their interactions with hub genes.

Supplementary Table 6 The microRNAs and their interactions with hub genes.
